# A Systematic Review of 10 Years of Augmented Reality Usability Studies: 2005 to 2014

**DOI:** 10.3389/frobt.2018.00037

**Published:** 2018-04-17

**Authors:** Arindam Dey, Mark Billinghurst, Robert W. Lindeman, J. Edward Swan

**Affiliations:** ^1^Empathic Computing Laboratory, University of South Australia, Mawson Lakes, SA, Australia; ^2^Human Interface Technology Lab New Zealand (HIT Lab NZ), University of Canterbury, Christchurch, New Zealand; ^3^Mississippi State University, Starkville, MS, United States

**Keywords:** augmented reality, systematic review, user studies, usability, experimentation, classifications

## Abstract

Augmented Reality (AR) interfaces have been studied extensively over the last few decades, with a growing number of user-based experiments. In this paper, we systematically review 10 years of the most influential AR user studies, from 2005 to 2014. A total of 291 papers with 369 individual user studies have been reviewed and classified based on their application areas. The primary contribution of the review is to present the broad landscape of user-based AR research, and to provide a high-level view of how that landscape has changed. We summarize the high-level contributions from each category of papers, and present examples of the most influential user studies. We also identify areas where there have been few user studies, and opportunities for future research. Among other things, we find that there is a growing trend toward handheld AR user studies, and that most studies are conducted in laboratory settings and do not involve pilot testing. This research will be useful for AR researchers who want to follow best practices in designing their own AR user studies.

## 1. Introduction

Augmented Reality (AR) is a technology field that involves the seamless overlay of computer generated virtual images on the real world, in such a way that the virtual content is aligned with real world objects, and can be viewed and interacted with in real time (Azuma, 1997). AR research and development has made rapid progress in the last few decades, moving from research laboratories to widespread availability on consumer devices. Since the early beginnings in the 1960's, more advanced and portable hardware has become available, and registration accuracy, graphics quality, and device size have been largely addressed to a satisfactory level, which has led to a rapid growth in the adoption of AR technology. AR is now being used in a wide range of application domains, including Education (Furió et al., 2013; Fonseca et al., 2014a; Ibáñez et al., 2014), Engineering (Henderson and Feiner, 2009; Henderson S. J. and Feiner, 2011; Irizarry et al., 2013), and Entertainment (Dow et al., 2007; Haugstvedt and Krogstie, 2012; Vazquez-Alvarez et al., 2012). However, to be widely accepted by end users, AR usability and user experience issues still need to be improved.

To help the AR community improve usability, this paper provides an overview of 10 years of AR user studies, from 2005 to 2014. Our work builds on the previous reviews of AR usability research shown in Table 1. These years were chosen because they cover an important gap in other reviews, and also are far enough from the present to enable the impact of the papers to be measured. Our goals are to provide a broad overview of user-based AR research, to help researchers find example papers that contain related studies, to help identify areas where there have been few user studies conducted, and to highlight exemplary user studies that embody best practices. We therefore hope the scholarship in this paper leads to new research contributions by providing outstanding examples of AR user studies that can help current AR researchers.

**Table 1:** Summary of earlier surveys of AR usability studies.

**Publication**	**Venues considered**	**Coverage years**	**Total reviewed publications**
Swan and Gabbard, 2005	IEEE ISMAR, ISWC,	1992–2004	21
	IEEE VR, and Presence		
Dünser et al., 2008	All venues in IEEE Xplore, ACM Digital Library, and Springer Link	1992–2007	165
Bai and Blackwell, 2012	IEEE ISMAR	2001–2010	71
This survey [2017]	All venues indexed in Scopus	2005–2014	291

### 1.1. Previous user study survey papers

Expanding on the studies shown in Table 1, Swan and Gabbard (2005) conducted the first comprehensive survey of AR user studies. They reviewed 1,104 AR papers published in four important venues between 1992 and 2004; among these papers they found only 21 that reported formal user studies. They classified these user study papers into three categories: (1) low-level perceptual and cognitive issues such as depth perception, (2) interaction techniques such as virtual object manipulation, and (3) collaborative tasks. The next comprehensive survey was by Dünser et al. (2008), who used a list of search queries across several common bibliographic databases, and found 165 AR-related publications reporting user studies. In addition to classifying the papers into the same categories as Swan and Gabbard (2005), they additionally classified the papers based on user study methods such as objective, subjective, qualitative, and informal. In another literature survey, Bai and Blackwell (2012) reviewed 71 AR papers reporting user studies, but they only considered papers published in the International Symposium on Mixed and Augmented Reality (ISMAR) between 2001 and 2010. They also followed the classification of Swan and Gabbard (2005), but additionally identified a new category of studies that investigated user experience (UX) issues. Their review thoroughly reported the evaluation goals, performance measures, UX factors investigated, and measurement instruments used. Additionally, they also reviewed the demographics of the studies' participants. However there has been no comprehensive study since 2010, and none of these earlier studies used an impact measure to determine the significance of the papers reviewed.

#### 1.1.1. Survey papers of AR subsets

Some researchers have also published review papers focused on more specific classes of user studies. For example, Kruijff et al. (2010) reviewed AR papers focusing on the perceptual pipeline, and identified challenges that arise from the environment, capturing, augmentation, display technologies, and user. Similarly, Livingston et al. (2013) published a review of user studies in the AR X-ray vision domain. As such, their review deeply analyzed perceptual studies in a niche AR application area. Finally, Rankohi and Waugh (2013) reviewed AR studies in the construction industry, although their review additionally considers papers without user studies. In addition to these papers, many other AR papers have included literature reviews which may include a few related user studies such as Wang et al. (2013), Carmigniani et al. (2011), and Papagiannakis et al. (2008).

### 1.2. Novelty and contribution

These reviews are valued by the research community, as shown by the number of times they have been cited (e.g., 166 Google Scholar citations for Dünser et al., 2008). However, due to a numebr of factors there is a need for a more recent review. Firstly, while early research in AR was primarily based on head-mounted displays (HMDs), in the last few years there has been a rapid increase in the use of handheld AR devices, and more advanced hardware and sensors have become available. These new wearable and mobile devices have created new research directions, which have likely impacted the categories and methods used in AR user studies. In addition, in recent years the AR field has expanded, resulting in a dramatic increase in the number of published AR papers, and papers with user studies in them. Therefore, there is a need for a new categorization of current AR user research, as well as the opportunity to consider new classification measures such as paper impact, as reviewing all published papers has become less plausible. Finally, AR papers are now appearing in a wider range of research venues, so it is important to have a survey that covers many different journals and conferences.

#### 1.2.1. New contributions over existing surveys

Compared to these earlier reviews, there are a number of important differences with the current survey, including:

- we have considered a larger number of publications from a wide range of sources
- our review covers more recent years than earlier surveys
- we have used paper impact to help filter the papers reviewed
- we consider a wider range of classification categories
- we also review issues experienced by the users.

#### 1.2.2. New aims of this survey

To capture the latest trends in usability research in AR, we have conducted a thorough, systematic literature review of 10 years of AR papers published between 2005 and 2014 that contain a user study. We classified these papers based on their application areas, methodologies used, and type of display examined. Our aims are to:

1. identify the primary application areas for user research in AR
2. describe the methodologies and environments that are commonly used
3. propose future research opportunities and guidelines for making AR more user friendly.

The rest of the paper is organized as follows: section 2 details the method we followed to select the papers to review, and how we conducted the reviews. Section 3 then provides a high-level overview of the papers and studies, and introduces the classifications. The following sections report on each of the classifications in more detail, highlighting one of the more impactful user studies from each classification type. Section 5 concludes by summarizing the review and identifying opportunities for future research. Finally, in the appendix we have included a list of all papers reviewed in each of the categories with detailed information.

## 2. Methodology

We followed a systematic review process divided into two phases: the search process and the review process.

### 2.1. Search process

One of our goals was to make this review as inclusive as practically possible. We therefore considered all papers published in conferences and journals between 2005 and 2014, which include the term “Augmented Reality,” and involve user studies. We searched the Scopus bibliographic database, using the same search terms that were used by Dünser et al. (2008) (Table 2). This initial search resulted in a total of 1,147 unique papers. We then scanned each one to identify whether or not it actually reported on AR research; excluding papers not related to AR reduced the number to 1,063. We next removed any paper that did not actually report on a user study, which reduced our pool to 604 papers. We then examined these 604 papers, and kept only those papers that provided all of the following information: (i) participant demographics (number, age, and gender), (ii) design of the user study, and (iii) the experimental task. Only 396 papers satisfied all three of these criteria. Finally, unlike previous surveys of AR usability studies, we next considered how much impact each paper had, to ensure that we were reviewing papers that others had cited. For each paper we used Google Scholar to find the total citations to date, and calculated its Average Citation Count (ACC):

(1) $$\begin{array}{l} \text{ACC}=\frac{\text{total lifetime citations}}{\text{lifetime }(\text{years})} \end{array}$$

For example, if a paper was published in 2010 (a 5 year lifetime until 2014) and had a total of 10 citations in Google Scholar in April 2015, its ACC would be 10/5 = 2.0. Based on this formula, we included all papers that had an ACC of at least 1.5, showing that they had at least a moderate impact in the field. This resulted in a final set of 291 papers that we reviewed in detail. We deliberately excluded papers more recent than 2015 because most of these hadn't gather significant citations yet.

**Table 2:** Search terms used in the Scopus database.

“Augmented reality” AND “user evaluation(s)”
“Augmented reality” AND “user study/-ies”
“Augmented reality” AND “feedback”
“Augmented reality” AND “experiment(s)”
“Augmented reality” AND “pilot study”
“Augmented reality” AND participant AND study
“Augmented reality” AND participant AND experiment
“Augmented reality” AND subject AND study
“Augmented reality” AND subject AND experiment

*We searched in Title, Abstract, and Keywords fields*.

### 2.2. Reviewing process

In order to review this many papers, we randomly divided them among the authors for individual review. However, we first performed a norming process, where all of the authors first reviewed the same five randomly selected papers. We then met to discuss our reviews, and reached a consensus about what review data would be captured. We determined that our reviews would focus on the following attributes:

- application areas and keywords
- experimental design (within-subjects, between-subjects, or mixed-factorial)
- type of data collected (qualitative or quantitative)
- participant demographics (age, gender, number, etc.)
- experimental tasks and environments
- type of experiment (pilot, formal, field, heuristic, or case study)
- senses augmented (visual, haptic, olfactory, etc.)
- type of display used (handheld, head-mounted display, desktop, etc.).

In order to systematically enter this information for each paper, we developed a Google Form. During the reviews we also flagged certain papers for additional discussion. Overall, this reviewing phase encompassed approximately 2 months. During this time, we regularly met and discussed the flagged papers; we also clarified any concerns and generally strove to maintain consistency. At the end of the review process we had identified the small number of papers where the classification was unclear, so we held a final meeting to arrive at a consensus view.

### 2.3. Limitations and validity concerns

Although we strove to be systematic and thorough as we selected and reviewed these 291 papers, we can identify several limitations and validity concerns with our methods. The first involves using the Scopus bibliographic database. Although using such a database has the advantage of covering a wide range of publication venues and topics, and although it did cover all of the venues where the authors are used to seeing AR research, it remains possible that Scopus missed publication venues and papers that should have been included. Second, although the search terms we used seem intuitive (Table 2), there may have been papers that did not use “Augmented Reality” as a keyword when describing an AR experience. For example, some papers may have used the term “Mixed Reality,” or “Artificial Reality.”

Finally, although using the ACC as a selection factor narrowed the initial 604 papers to 291, it is possible that the ACC excluded papers that should have been included. In particular, because citations are accumulated over time, it is quite likely that we missed some papers from the last several years of our 10-year review period that may soon prove influential.

## 3. High-level overview of reviewed papers

Overall, the 291 papers report a total of 369 studies. Table 3 gives summary statistics for the papers, and Table 4 gives summary statistics for the studies. These tables contain bar graphs that visually depict the magnitude of the numbers; each color indicates the number of columns are spanned by the bars. For example, in Table 3 the columns Paper, Mean ACC, and Mean Author Count are summarized individually, and the longest bar in each column is scaled according to the largest number in that column. However, Publications spans two columns, and the largest value is 59, and so all of the other bars for Publications are scaled according to 59.

**Table 3:**
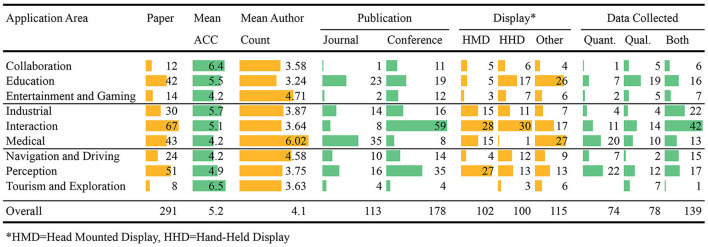
Summary of the 291 reviewed papers.

**Table 4:**
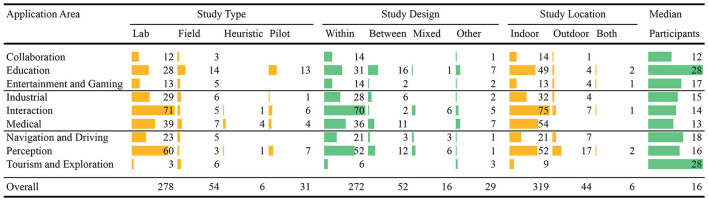
Summary of the 369 user studies reported by the 291 reviewed papers.

**Figure 1:**
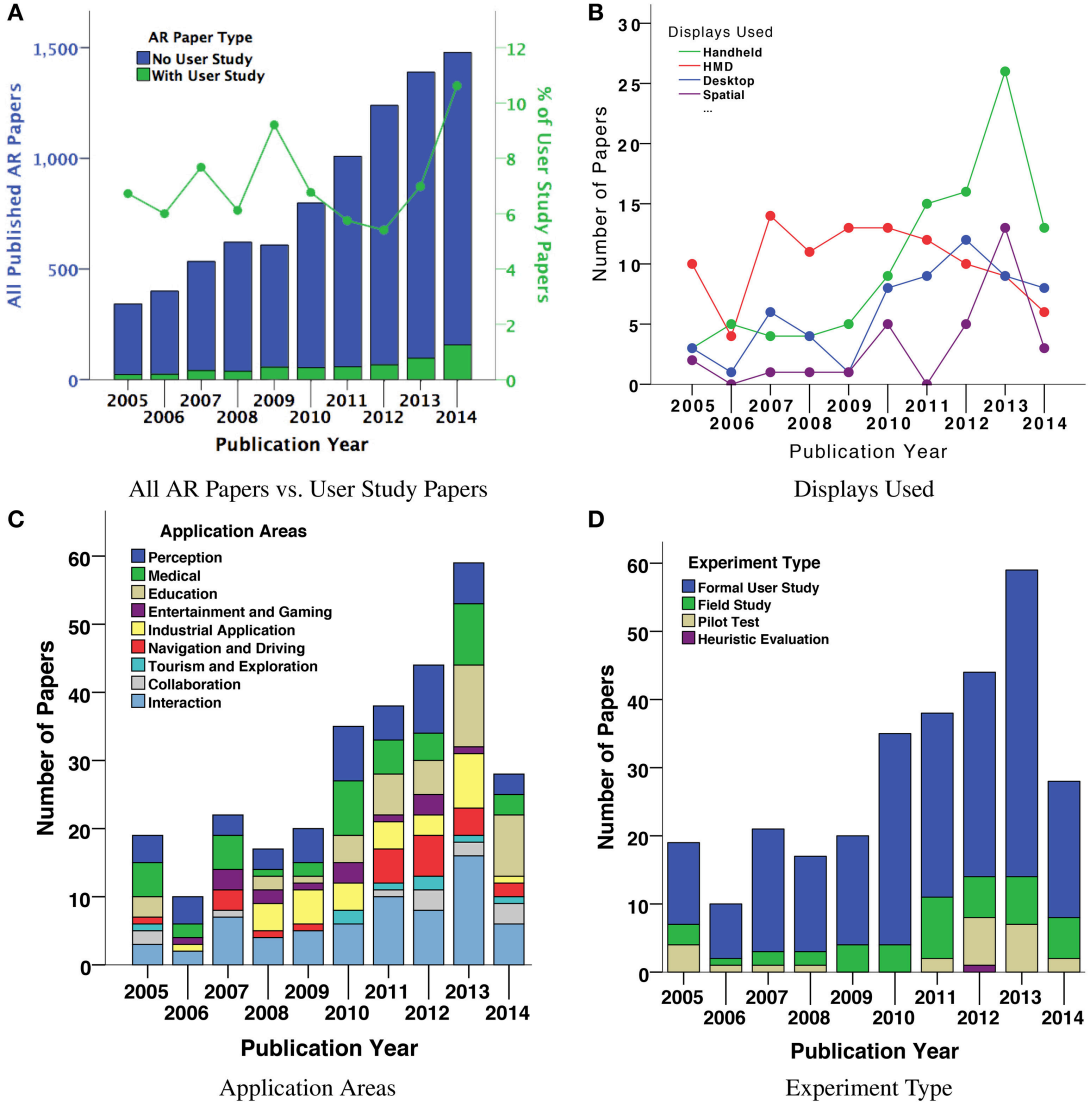
Throughout the 10 years, less than 10% of all published AR papers had a user study **(A)**. Out of the 291 reviewed papers, since 2011 most papers have examined handheld displays, rather than HMDs **(B)**. We filtered the papers based on ACC and categorized them into nine application areas; the largest areas are Perception and Interaction **(C)**. Most of the experiments were in controlled laboratory environments **(D)**.

Figure 1 further summarizes the 291 papers through four graphs, all of which indicate changes over the 10 year period between 2005 and 2014. Figure 1A shows the fraction of the total number of AR papers that report user studies, Figure 1B analyzes the kind of display used, Figure 1C categorizes the experiments into application areas, and Figure 1D categorizes the papers according to the kind of experiment that was conducted.

### 3.1. Fraction of user studies over time

Figure 1A shows the total number of AR papers published between 2005 and 2014, categorized by papers with and without a user study. As the graph shows, the number of AR papers published in 2014 is five times that published in 2005. However, the proportion of user study papers among all AR papers has remained low, less than 10% of all publication for each year.

### 3.2. Study design

As shown in Table 4, most of the papers (213, or 73%) used a within-subjects design, 43 papers (15%) used a between-subjects design, and 12 papers (4%) used a mixed-factorial design. However, there were 23 papers (8%) which used different study designs than the ones mentioned above, such as Baudisch et al. (2013), Benko et al. (2014), and Olsson et al. (2009).

### 3.3. Study type

We found that it was relatively rare for researchers to report on conducting pilot studies before their main study. Only 55 papers (19%) reported conducting at least one pilot study in their experimentation process and just 25 of them reported the pilot studies with adequate details such as study design, participants, and results. This shows that the importance of pilot studies is not well recognized. The majority of the papers (221, or 76%) conducted the experiments in controlled laboratory environments, while only 44 papers (15%) conducted the experiments in a natural environment or as a field study (Figure 1D). This shows a lack of experimentation in real world conditions. Most of the experiments were formal user studies, and there were almost no heuristic studies, which may indicate that the heuristics of AR applications are not fully developed and there exists a need for heuristics and standardization.

### 3.4. Data type

In terms of data collection, a total of 139 papers (48%) collected both quantitative and qualitative data, 78 (27%) papers only qualitative, and 74 (25%) only quantitative. For the experimental task, we found that the most popular task involved performance (178, or 61%), followed by filling out questionnaires (146, or 50%), perceptual tasks (53, or 18%), interviews (41, or 14%) and collaborative tasks (21, or 7%). In terms of dependent measures, subjective ratings were the most popular with 167 papers (57%), followed by error/accuracy measures (130, or 45%), and task completion time (123, or 42%). We defined task as any activity that was carried out by the participants to provide data—both quantitative and/or qualitative—about the experimental system(s). Note that many experiments used more than one experimental task or dependent measure, so the percentages sum to more than 100%. Finally, the bulk of the user studies were conducted in an indoor environment (246, or 83%), not outdoors (43, or 15%), or a combination of both settings (6, or 2%).

### 3.5. Senses

As expected, an overwhelming majority of papers (281, or 96%) augmented the visual sense. Haptic and Auditory senses were augmented in 27 (9%) and 21 (7%) papers respectively. Only six papers (2%) reported augmenting only the auditory sense and five (2%) papers reported augmenting only the haptic sense. This shows that there is an opportunity for conducting more user studies exploring non-visual senses.

### 3.6. Participants

The demographics of the participants showed that most of the studies were run with young participants, mostly university students. A total of 182 papers (62%) used participants with an approximate mean age of less than 30 years. A total of 227 papers (78%) reported involving female participants in their experiments, but the ratio of female participants to male participants was low (43% of total participants in those 227 papers). When all 291 papers are considered only 36% of participants were females. Many papers (117, or 40%) did not explicitly mention the source of participant recruitment. From those that did, most (102, or 35%) sourced their participants from universities, whereas only 36 papers (12%) mentioned sourcing participants from the general public. This shows that many AR user studies use young male university students as their subjects, rather than a more representative cross section of the population.

### 3.7. Displays

We also recorded the displays used in these experiments (Table 3). Most of the papers used either HMDs (102 papers, or 35%) or handhelds (100 papers, or 34%), including six papers that used both. Since 2009, the number of papers using HMDs started to decrease while the number of papers using handheld displays increased (Figure 1B). For example, between 2010 and 2014 (204 papers in our review), 50 papers used HMDs and 79 used handhelds, including one paper that used both, and since 2011 papers using handheld displays consistently outnumbered papers using HMDs. This trend—that handheld mobile AR has recently become the primary display for AR user studies—is of course driven by the ubiquity of smartphones.

### 3.8. Categorization

We categorized the papers into nine different application areas (Tables 3, 4): (i) Perception (51 papers, or 18%), (ii) Medical (43, or 15%), (iii) Education (42, or 14%), (iv) Entertainment and Gaming (14, or 5%), (v) Industrial (30, or 10%), (vi) Navigation and Driving (24, or 9%), (vii) Tourism and Exploration (8, or 2%), (viii) Collaboration (12, or 4%), and (ix) Interaction (67, or 23%). Figure 1C shows the change over time in number of AR papers with user studies in these categories. The Perception and Interaction categories are rather general areas of AR research, and contain work that reports on more low-level experiments, possibly across multiple application areas. Our analysis shows that there are fewer AR user studies published in Collaboration, Tourism and Exploration, and Entertainment and Gaming, identifying future application areas for user studies. There is also a noticeable increase in the number of user studies in educational applications over time. The drop in number of papers in 2014 is due to the selection criteria of papers having at least 1.5 average citations per year, as these papers were too recent to be cited often. Interestingly, although there were relatively few of them, papers in Collaboration, Tourism and Exploration categories received noticeably higher ACC scores than other categories.

### 3.9. Average authors

As shown in Table 3, most categories had a similar average number of authors for each paper, ranging between 3.24 (Education) and 3.87 (Industrial). However papers in the Medical domain had the highest average number of authors (6.02), which indicates the multidisciplinary nature of this research area. In contrast to all other categories, most of the papers in the Medical category were published in journals, compared to the common AR publications venues, which are mostly conferences. Entertainment and Gaming (4.71), and Navigation and Driving (4.58) also had considerably higher numbers of authors per paper on average.

### 3.10. Individual studies

While a total of 369 studies were reported in these 291 papers (Table 4), the majority of the papers (231, or 80%) reported only one user study. Forty-seven (16.2%), nine (3.1%), two (<1%), and one (<1%) papers reported two, three, four, and five studies respectively, including pilot studies. In terms of the number of participants used (median) in each study, Tourism and Exploration, and Education were the highest among all categories with an average of 28 participants per study. Other categories used between 12 and 18 participants per study, while the overall median stands at 16 participants. Based on this insight, it can be claimed that 12 to 18 participants per study is a typical range in the AR community. Out of the 369 studies 31 (8.4%) were pilot studies, six (1.6%) heuristic evaluation, 54 (14.6%) field studies, and rest of the 278 (75.3%) were formal controlled user studies. Most of the studies (272, or 73.7%) were designed as within-subjects, 52 (14.1%) between-subjects, and 16 (4.3%) as mixed-factors (Table 4).

In the following section we review user studies in each of the nine application areas separately. We provide a commentary on each category and also discuss a representative paper with the highest ACCs in each application area, so that readers can understand typical user studies from that domain. We present tables summarizing all of the papers from these areas at the end of the paper.

## 4. Application areas

### 4.1. Collaboration

A total of 15 studies were reported in 12 papers in the Collaboration application area. The majority of the studies investigated some form of remote collaboration (Table 5), although Henrysson et al. (2005a) presented a face-to-face collaborative AR game. Interestingly, out of the 15 studies, eight reported using handheld displays, seven used HMDs, and six used some form of desktop display. This makes sense as collaborative interfaces often require at least one collaborator to be stationary and desktop displays can be beneficial in such setups. One noticeable feature was the low number of studies performed in the wild or in natural settings (field studies). Only three out of 15 studies were performed in natural settings and there were no pilot studies reported, which is an area for potential improvement. While 14 out of 15 studies were designed to be within-subjects, only 12 participants were recruited per study. On average, roughly one-third of the participants were females in all studies considered together. All studies were performed in indoor locations except for (Gauglitz et al., 2014b), which was performed in outdoors. While a majority of the studies (8) collected both objective (quantitative) and subjective (qualitative) data, five studies were based on only subjective data, and two studies were based on only objective data, both of which were reported in one paper (Henrysson et al., 2005a). Besides subjective feedback or ratings, task completion time and error/accuracy were other prominent dependent variables used. Only one study used NASA TLX (Wang and Dunston, 2011).

**Table 5:** Summary of user studies in Collaboration application area.

**References**	**Topic**	**Data type**	**Displays used**	**Dependent variables**	**Study type**	**Participants (female)**
Almeida et al., 2012	AR based video meetings	S	DT	Rating	Formal	10 (0)
Chastine et al., 2007	Collaboration	S	HMD	Interview answers	Formal	16 (4)
Chen et al., 2013	Remote collaboration	O + S	HH	Time, Subjective feedback	Field	16 (7)
Gauglitz et al., 2012	Remote collaboration	O + S	HH, DT	Error/Accuracy, Rating	Formal	48 (21)
	with an expert				Completed task count	
Gauglitz et al., 2014a	Annotations in	S	HH, DT,	User preference	Field	11 (5)
	remote Collaboration		DT touchscreen			
Gauglitz et al., 2014b	Remote collaboration	O + S	HH, DT	Time, Error/Accuracy, Rating	Formal	60 (29)
Grasset et al., 2005	Collaboration	O + S	HMD	Time, Error/Accuracy,	Formal	14 (2)
				Rating, Subject movement		
Henrysson et al., 2005a	Games, Interaction,	O	HH	Rating	Formal	12 (0)
	Tangible Interfaces					
Kasahara and Rekimoto, 2014	Remote Collaboration	O + S	HMD	Time, Rating, Body movement	Formal	10 (0)
Poelman et al., 2012	Remote Collaboration,	S	HMD	Observation and discussion	Field	5 (0)
	Crime Scene Investigation					
Sodhi et al., 2013	Remote Collaboration	S	HH	Rating	Formal	8 (1)
Wang and Dunston, 2011	Collaboration	O + S	HMD	Time, NASA TLX	Formal	16 (4)

*S, Subjective; O, Objective; DT, Desktop; HH, handheld. Participant numbers are absolute values, and where more than one study was reported in the paper we used average counts*.

#### 4.1.1. Representative paper

As an example of the type of collaborative AR experiments conducted, we discuss the paper of Henrysson et al. (2005a) in more detail. They developed an AR-based face-to-face collaboration tool using a mobile phone and reported on two user studies. This paper received an ACC of 22.9, which is the highest in this category of papers. In the first study, six pairs of participants played a table-top tennis game in three conditions—face to face AR, face to face non-AR, and non-face to face collaboration. In the second experiment, the authors added (and varied) audio and haptic feedback to the games and only evaluated face to face AR. The same six pairs were recruited for this study as well. Authors collected both quantitative and qualitative (survey and interview) data, although they focused more on the latter. They asked questions regarding the usability of system and asked participants to rank the conditions. They explored several usability issues and provided design guidelines for developing face to face collaborative AR applications using handheld displays. For example, designing applications that have a focus on a single shared work space.

#### 4.1.2. Discussion

The work done in this category is mostly directed toward remote collaboration. With the advent of modern head mounted devices such the Microsoft HoloLens, new types of collaborations can be created, including opportunities for enhanced face to face collaboration. Work needs to be done toward making AR-based remote collaboration akin to the real world with not only shared understanding of the task but also shared understanding of the other collaborators emotional and physiological states. New gesture-based and gaze-based interactions and collaboration across multiple platforms (e.g., between AR and virtual reality users) are novel future research directions in this area.

### 4.2. Education

Fifty-five studies were reported in 42 papers in the Education application area (Table 6). As expected, all studies reported some kind of teaching and learning applications, with a few niche areas, such as music training, educational games, and teaching body movements. Out of 55 studies, 24 used handheld displays, 8 used HMDs, 16 used some form of desktop displays, and 11 used spatial or large-scale displays. One study had augmented only sound feedback and used a head-mounted speaker (Hatala and Wakkary, 2005). Again, a trend of using handheld displays is prominent in this application area as well. Among all the studies reported, 13 were pilot studies, 14 field studies, and 28 controlled lab-based experiments. Thirty-one studies were designed as within-subjects studies, and 16 as between-subjects. Six studies had only one condition tested. The median number of participants was 28, jointly highest among all application areas. Almost 43% of participants were females. Forty-nine studies were performed in indoor locations, four in outdoor locations, and two studies were performed in both locations. Twenty-five studies collected only subjective data, 10 objective data, and 20 studies collected both types of data. While subjective rating was the primary dependent measure used in most of the studies, some specific measures were also noticed, such as pre- and post-test scores, number of items remembered, and engagement. From the keywords used in the papers, it appears that *learning* was the most common keyword and *interactivity, users*, and *environments* also received noticeable importance from the authors.

**Table 6:** Summary of user studies in Education application area.

**References**	**Topic**	**Data type**	**Displays used**	**Dependent measures**	**Study type**	**Participants (female)**
Anderson and Bischof, 2014	Training, Learning	O + S	DT	IMI Score, Isolation, Muscle control	Formal	12 (6)
Arvanitis et al., 2009	Learning	O + S	HMD	Rating, Physiological measures, sense of welbeing	Formal	5 (2)
Asai et al., 2005	AR Instructions/Annotations	S	HMD, HH	Rating	Formal	22 (15)
Cai et al., 2013	Education	O + S	S/LS	Rating, Exam questions correct	Formal	50 (30)
Cai et al., 2014	Learning and Teaching	O + S	DT	Error/Accuracy, Rating	Formal	29 (13)
Chang et al., 2013	Training	O + S	S/LS	Error/Accuracy, Rating	Formal	3 (1)
Chiang et al., 2014	Education	O	HH	Learning outcomes	Field	57 (NA)
Cocciolo and Rabina, 2013	Learning, Tourism	S	HH	Interview response	Field	34 (74)
Dünser et al., 2012b	Education	O	HH	Error/Accuracy	Pilot	10 (10)
Fonseca et al., 2014b	Education	O + S	HH	Rating, Learning test scores	Formal	48 (18)
Fonseca et al., 2014a	Education	S	HH, DT	Rating	Field	57 (29)
Freitas and Campos, 2008	Educational AR Game for	O + S	S/LS, DT	Error/Accuracy	Field	54 (32)
	2nd Graders (7-8 years old)			Qualitative Observation		
Furió et al., 2013	Education	S	Unspecified	Rating, Multi-choice question responses	Pilot	117 (74)
Gama et al., 2012	AR for movement training	S	DT	Rating	Pilot	10 (NA)
Hatala and Wakkary, 2005	Museum guide	O + S	Head-mounted speakers	Rating, Subjective response	Field	8 (4)
Hou and Wang, 2013	Training	O	DT	Time, Error/Accuracy	Formal	28 (14)
Hsiao, 2010	Education	O + S	S/LS	Rating	Formal	673 (338)
Hsiao et al., 2012	E-learning	O + S	S/LS, DT	Error/Accuracy, Rating	Formal	884 (489)
Hunter et al., 2010	Educational AR	S	Tangible, location-aware	Observation	Field	9 (NA)
		S	blocks with a video screen			
Ibáñez et al., 2014	Education	O + S	HH	Rating, knowledge learnt	Formal	60 (15)
				- pre and post test		
Iwata et al., 2011	Self-learning, Gaming	S	DT	Rating	Formal	18 (1)
Juan et al., 2011b	AR handheld gaming,	S	HH	Rating	Formal	38 (14)
	Educational gaming					
Juan et al., 2011a	AR educational game	S	DT	Rating, knowledge of animals	Formal	31 (14)
Kurt, 2010	Learning	S	HH	Rating, field notes, observations	Field	55 (NA)
Li et al., 2011	AR for education	O + S	HH	Error/Accuracy, Rating	Formal	36 (20)
Liarokapis, 2005	Music Education	S	HMD, DT	Ease of use/usability	Pilot	9 (NA)
Lin et al., 2013	Education	O	HH	Correct answers in physics tests	Formal	40 (25)
Luckin and Fraser, 2011	Learning and Teaching	O + S	DT	Rating, Items remembered, Engagement	Field	304 (NA)
Martin-Gutierrez, 2011	AR use in the classroom	S	DT	Rating	Formal	47 (NA)
Oh and Byun, 2012	Interactive learning systems	S	HH	Rating	Pilot	15 (6)
Salvador-Herranz et al., 2013	Education	S	S/LS	Rating	Pilot	21 (9)
Santos et al., 2013	AR X-Ray techniques for education	S	HH	Rating	Pilot	27.3 (15.6)
Schwerdtfeger and Klinker, 2008	AR enabled instructions	O + S	HMD	Time, Error/Accuracy,	Formal	24 (10)
				Subjective feedback		
Shatte et al., 2014	Library management	O	HH	Time	Formal	35 (NA)
Sommerauer and Müller, 2014	Education	O	HH	Error/Accuracy, Rating	Field	101 (39)
Sumadio and Rambli, 2010	Education	O + S	DT	Rating	Formal	33 (20)
Szymczak et al., 2012	Multi-sensory AR	S	HH	Rating	Field	17 (10.5)
	for historic city sites					
Toyama et al., 2013	Reading Assistance	O	HMD	Error/Accuracy	Pilot	12 (NA)
Weing et al., 2013	Music Education	S	S/LS	Interview questions	Pilot	4 (0)
Wojciechowski and Cellary, 2013	Education	S	DT	Rating	Formal	42 (NA)
Yamabe and Nakajima, 2013	Training	S	S/LS, DT	Rating	Formal	10 (1.5)
Zhang et al., 2014	Teaching	O + S	HH	Error/Accuracy, flow experience	Field	147 (54)

*S, Subjective; O, Objective; DT, Desktop; HH, handheld. Participant numbers are absolute values and where more than one studies were reported in the paper we used average counts*.

#### 4.2.1. Representative paper

The paper from Fonseca et al. (2014a) received the highest ACC (22) in the Education application area of AR. They developed a mobile phone-based AR teaching tool for 3D model visualization and architectural projects for classroom learning. They recruited a total of 57 students (29 females) in this study and collected qualitative data through questionnaires and quantitative data through pre- and post-tests. This data was collected over several months of instruction. The primary dependent variable was the academic performance improvement of the students. Authors used five-point Likert-scale questions as the primary instrument. They reported that using the AR tool in the classroom was correlated with increased motivation and academic achievement. This type of longitudinal study is not common in the AR literature, but is helpful in measuring the actual real-world impact of any application or intervention.

#### 4.2.2. Discussion

The papers in this category covered a diverse range of education and training application areas. There are some papers used AR to teach physically or cognitively impaired patients, while a couple more promoted physical activity. This set of papers focused on both objective and subjective outcomes. For example, Anderson and Bischof (2014) reported a system called ARM trainer to train amputees in the use of myoelectric prostheses that provided an improved user experience over the current standard of care. In a similar work, Gama et al. (2012) presented a pilot study for upper body motor movements where users were taught to move body parts in accordance to the instructions of an expert such as physiotherapist and showed that AR-based system was preferred by the participants. Their system can be applied to teach other kinds of upper body movements beyond just rehabilitation purposes. In another paper, Chang et al. (2013) reported a study where AR helped cognitively impaired people to gain vocational job skills and the gained skills were maintained even after the intervention. Hsiao et al. (2012) and Hsiao (2010) presented a couple of studies where physical activity was included in the learning experience to promote “learning while exercising". There are few other papers that gamified the AR learning content and they primarily focused on subjective data. Iwata et al. (2011) presented ARGo an AR version of the GO game to investigate and promote self-learning. Juan et al. (2011b) developed ARGreenet game to create awareness for recycling. Three papers investigated education content themed around tourism and mainly focused on subjective opinion. For example, Hatala and Wakkary (2005) created a museum guide educating users about the objects in the museum and Szymczak et al. (2012) created multi-sensory application for teaching about the historic sites in a city. There were several other papers that proposed and evaluated different pedagogical approaches using AR including two papers that specifically designed for teaching music such as Liarokapis (2005) and Weing et al. (2013). Overall these papers show that in the education space a variety of evaluation methods can be used, focusing both on educational outcomes and application usability. Integrating methods of intelligent tutoring systems (Anderson et al., 1985) with AR could provide effective tools for education. Another interesting area to explore further is making these educational interfaces adaptive to the users cognitive load.

### 4.3. Entertainment and gaming

We reviewed a total of 14 papers in the Entertainment and Gaming area with 18 studies were reported in these papers (Table 7). A majority of the papers reported a gaming application while fewer papers reported about other forms of entertainment applications. Out of the 18 studies, nine were carried out using handheld displays and four studies used HMDs. One of the reported studies, interestingly, did not use any display (Xu et al., 2011). Again, the increasing use of handheld displays is expected as this kind of display provides greater mobility than HMDs. Five studies were conducted as field studies and the rest of the 13 studies were controlled lab-based experiments. Fourteen studies were designed as within-subjects and two were between-subjects. The median number of participants in these studies was 17. Roughly 41.5% of participants were females. Thirteen studies were performed in indoor areas, four were in outdoor locations, and one study was conducted in both locations. Eight studies collected only subjective data, another eight collected both subjective and objective data, and the remaining two collected only objective data. Subjective preference was the primary measure of interest. However, task completion time was also another important measure. In this area, error/accuracy was not found to be a measure in the studies used. In terms of the keywords used by the authors, besides games, mobile and handheld were other prominent keywords. These results highlight the utility of handheld displays for AR Entertainment and Gaming studies.

**Table 7:** Summary of user studies in Entertainment and Gaming application area.

**References**	**Topic**	**Data type**	**Displays used**	**Dependent measures**	**Study type**	**Participants (female)**
Baudisch et al., 2013	Gaming	S	Audio interface	Rating	Formal	30 (7)
Dow et al., 2007	Entertainment	O + S	HMD	Time, Observations, subject interviews	Formal	12 (6)
Grubert et al., 2012	Mobile gaming	O + S	HH	Time, Rating, Fatigue	Formal	16 (8)
				(as evidenced by phone posture)		
Haugstvedt and Krogstie, 2012	Mobile AR for	S	HH	Rating	Field	121 (60.5)
	Cultural Heritage					
Henze and Boll, 2010a	Mobile music listening	S	HH	Rating	Formal	15 (3.5)
Kern et al., 2006	Gaming	S	DT	Subjective opinion	Formal	3 (2)
Mulloni et al., 2008	Handheld AR Gaming	S	HH	Rating	Field	12 (6)
Oda and Feiner, 2009	Handheld AR gaming	O + S	HH	Rating, Distance	Formal	18 (3)
Schinke et al., 2010	AR tourism information	O	HH	Error/Accuracy	Formal	26 (13)
	systems, outdoor AR					
Vazquez-Alvarez et al., 2012	Tourism, Navigation	O + S	Headphone	Time, Rating, Distance covered,	Field	8 (2)
				Walking speed, Times stopped		
Wither et al., 2010	AR story-telling	O + S	HH	Rating, Subjective feedback	Formal	16 (9)
Xu et al., 2008	AR Gaming,	O + S	AR GamePad	Rating	Formal	18 (5)
	Collaboration		(Gizmondo)			
Xu et al., 2011	Handheld AR for	O	No displays used	Coding of recorded video	Field	9 (NA)
	social tabletop games					
Zhou et al., 2007	Gaming, Audio AR	O + S	HMD	Time, Error/Accuracy, Rating	Formal	40 (13)

*S, Subjective; O, Objective; DT, Desktop; HH, handheld. Participant numbers are absolute values and where more than one studies were reported in the paper we used average counts*.

#### 4.3.1. Representative paper

Dow et al. (2007) presented a qualitative user study exploring the impact of immersive technologies on presence and engagement, using interactive drama, where players had to converse with characters and manipulate objects in the scene. This paper received the highest ACC (9.5) in this category of papers. They compared two versions of desktop 3D based interfaces with an immersive AR based interface in a lab-based environment. Participants communicated in the desktop versions using keyboards and voice. The AR version used a video see-though HMD. They recruited 12 participants (six females) in the within-subjects study, each of whom had to experience interactive dramas. This paper is unusual because user data was collected mostly from open-ended interviews and observation of participant behaviors, and not task performance or subjective questions. They reported that immersive AR caused an increased level of user Presence, however, higher presence did not always led to more engagement.

#### 4.3.2. Discussion

It is clear that advances in mobile connectivity, CPU and GPU processing capabilities, wearable form factors, tracking robustness, and accessibility to commercial-grade game creation tools is leading to more interest in AR for entertainment. There is significant evidence from both AR and VR research of the power of immersion to provide a deeper sense of presence, leading to new opportunities for enjoyment in Mixed Reality (a continuum encompassing both AR and VR Milgram et al., 1995) spaces. Natural user interaction will be key to sustaining the use of AR in entertainment, as users will shy away from long term use of technologies that induce fatigue. In this sense, wearable AR will probably be more attractive for entertainment AR applications. In these types of entertainment applications, new types of evaluation measures will need to be used, as shown by the work of Dow et al. (2007).

### 4.4. Industrial

There was a total of 30 papers reviewed that focused on Industrial applications, and together they reported 36 user studies. A majority of the studies reported maintenance and manufacturing/assembly related tasks (Table 8). Eleven studies used handheld displays, 21 used HMDs, four used spatial or large screen displays, and two used desktop displays. The prevalence of HMDs was expected as most of the applications in this area require use of both hands at times, and as such HMDs are more suitable as displays. Twenty-nine studies were executed in a formal lab-based environment and only six studies were executed in their natural setups. We believe performing more industrial AR studies in the natural environment will lead to more-usable results, as controlled environments may not expose the users to the issues that they face in real-world setups. Twenty-eight studies were designed as within-subjects and six as between-subjects. One study was designed to collect exploratory feedback from a focus group (Olsson et al., 2009). The median number of participants used in these studies was 15 and roughly 23% of them were females. Thirty-two studies were performed in indoor locations and four in outdoor locations. Five studies were based on only subjective data, four on only objective data, and rest of the 27 collected both kinds of data. Use of NASA TLX was very common in this application area, which was expected given the nature of the tasks. Time and error/accuracy were other commonly used measurements along with subjective feedback. The keywords used by the authors to describe their papers highlight a strong interest in *interaction, interfaces*, and *users*. *Guidance* and *maintenance* are other prominent keywords that authors used.

**Table 8:** Summary of user studies in Industrial area.

**References**	**Topic**	**Data type**	**Displays used**	**Dependent measures**	**Study type**	**Participants (female)**
Allen et al., 2011	AR for architectural planning	S	HH	Rating	Formal	18 (7)
Bruno et al., 2010	Industrial prototyping	O + S	HMD	Error/Accuracy	Formal	30 (NA)
Bunnun et al., 2013	Modeling	O + S	HH	Time, Error/Accuracy, Rating	Formal	10 (3)
Fiorentino et al., 2013	None	O + S	HMD	Time, Error/Accuracy, Rating	Formal	14 (3)
Fiorentino et al., 2014	Maintenance	O + S	S/LS	Time, Error/Accuracy, Rating	Formal	14 (3)
Gavish et al., 2013	Industrial maintenance	O + S	HH	Time, Error/Accuracy	Formal	40 (1)
	and assembly			Rating, unsolved errors		
Hakkarainen et al., 2008	Object assembly	S	HH	Rating	Pilot	8 (NA)
Hartl et al., 2013	Document Verification	O + S	HH	Time, Error/Accuracy, Rating,	Formal	17 (1)
				Nasa TLX, AttrakDiff UX survey	Formal	
Henderson and Feiner, 2009	AR Maintenance	O + S	HMD, DT	Time, Rating	Field	6 (0)
Henderson and Feiner, 2010	Industrial AR	O + S	HMD	Time, Error/Accuracy, Rating	Formal	15 (4)
Henderson S. and Feiner, 2011	Maintenance and repair, Defense	O + S	HMD	Time, Error/Accuracy, Rating,	Formal	6 (0)
				Head movement, Supporting task focus		
Henderson S. J. and Feiner, 2011	AR for Industrial Tasks,	O + S	HMD	Time, Error/Accuracy, Rating	Formal	11.3 (2.3)
	Assembly Tasks					
Irizarry et al., 2013	Construction	O + S	HH	Time, Rating	Formal	30 (9)
Lau et al., 2012	Tangible UI, 3D modeling	O + S	HMD	Time, Error/Accuracy, Rating	Formal	10 (4)
Magnusson et al., 2010	Pointing in space	O	HH	Time, Error/Accuracy, Rating	Formal	6 (3)
Markov-Vetter et al., 2012	Flight	O + S	HMD	Time, Error/Accuracy,	Formal	6 (1)
				Rating, Pointing Behavior,		
				Physiological Measures, NASA RTLX		
Marner et al., 2013	None	O + S	S/LS	Time, Error/Accuracy, Rating	Formal	24 (6)
Olsson et al., 2009	Design	S	HH	Rating	Formal	23 (10)
Petersen and Stricker, 2009	Industrial assembly	O + S	S/LS	Time	Formal	15 (10)
Rauhala et al., 2006	Humidity data visualization	O + S	HH	Error/Accuracy	Formal	10 (3)
Reif and Günthner, 2009	Storage facility management	O + S	HMD	Time, Error/Accuracy, Rating	Formal	16 (3)
Rosenthal et al., 2010	AR for guiding manual tasks	O	S/LS	Time, Error/Accuracy	Formal	30 (17)
Schall et al., 2013a	Surveying	S	HH	Rating	Field	16 (4)
Schoenfelder and Schmalstieg, 2008	Industrial building acceptance	O + S	HMD, HH	Error/Accuracy, Navigational activity	Formal	36 (9)
Schwerdtfeger et al., 2009	Stock Picking	O + S	HMD	Time, Error/Accuracy,	Formal	13.5 (3.5)
				Rating, NASA TLX		
Schwerdtfeger et al., 2011	Order picking in a warehouse	O + S	HMD	Time, Error/Accuracy, Observation	Field	22.3 (10)
Tumler et al., 2008	Industrial AR; Order Picking Task	O	HMD	Rating, heart rate	Formal	12 (0)
Vignais et al., 2013	Ergonomics	O + S	HMD	Time, Rating, Articulation score	Formal	12 (0)
Yeh et al., 2012	Construction	O	Projected AR	Time, Error/Accuracy	Formal	34 (7)
Yuan et al., 2008	Assembly, manufacturing	O + S	HMD, DT	Time, Error/Accuracy	Formal	14 (4)

*S, Subjective; O, Objective; DT, Desktop; HH, handheld. Participant numbers are absolute values and where more than one studies were reported in the paper we used average counts*.

#### 4.4.1. Representative paper

As an example of the papers written in this area, Henderson S. and Feiner (2011) published a work exploring AR documentation for maintenance and repair tasks in a military vehicle, which received the highest ACC (26.25) in the Industrial area. They used a video see-though HMD to implement the study application. In the within-subjects study, the authors recruited six male participants who were professional military mechanics and they performed the tasks in the field settings. They had to perform 18 different maintenance tasks using three conditions—AR, LCD, and HUD. Several quantitative and qualitative (questionnaire) data were collected. As dependent variables they used task completion time, task localization time, head movement, and errors. The AR condition resulted in faster locating tasks and fewer head-movements. Qualitatively, AR was also reported to be more intuitive and satisfying. This paper provides an outstanding example of how to collect both qualitative and quantitative measures in an industrial setting, and so get a better indication of the user experience.

#### 4.4.2. Discussion

Majority of the work in this category focused on maintenance and assembly tasks, whereas a few investigated architecture and planning tasks. Another prominent line of work in this category is military applications. Some work also cover surveying and item selection (stock picking). It will be interesting to investigate non-verbal communication cues in collaborative industrial applications where people form multiple cultural background can easily work together. As most of the industrial tasks require specific training and working in a particular environment, we assert that there needs to be more studies that recruit participants from the real users and perform studies in the field when possible.

### 4.5. Interaction

There were 71 papers in the Interaction design area and 83 user studies reported in these papers (see Table 9). Interaction is a very general area in AR, and the topics covered by these papers were diverse. Forty studies used handheld displays, 33 used HMDs, eight used desktop displays, 12 used spatial or large-screen displays, and 10 studies used a combination of multiple display types. Seventy-one studies were conducted in a lab-based environment, five studies were field studies, and six were pilot studies. Jones et al. (2013) were the only authors to conduct a heuristic evaluation. The median number of participants used in these studies was 14, and approximately 32% of participants were females. Seventy-five studies were performed in indoor locations, seven in outdoor locations, and one study used both locations. Sixteen studies collected only subjective data, 14 collected only objective data, and 53 studies collected both types of data. Task completion time and error/accuracy were the most commonly used dependent variables. A few studies used the NASA TLX workload survey (Robertson et al., 2007; Henze and Boll, 2010b) and most of the studies used different forms of subjective ratings, such as ranking conditions and rating on a Likert scale. The keywords used by authors identify that the papers in general were focused on *interaction, interface, user, mobile*, and *display* devices.

**Table 9:** Summary of user studies in Interaction application area.

**References**	**Topic**	**Data type**	**Displays used**	**Dependent measures**	**Study type**	**Participants (female)**
Ajanki et al., 2011		O + S	HMD, HH	Error/Accuracy, Rating	Formal	7.5 (1.5)
Axholt et al., 2011	AR Optical See-Through Calibration	O	HMD	Error/Accuracy	Formal	11 (1)
Bai et al., 2012	Phone-based AR interaction methods	O + S	HH	Time, Error/Accuracy, Rating	Formal	10 (4)
Bai et al., 2013a	Handheld AR	O + S	HH	Time, Rating	Formal	32 (16)
Bai et al., 2014	Basic AR interaction methods	O + S	HMD	Time, Error/Accuracy, Rating	Formal	5 (0)
Baričević et al., 2012	Handheld AR	O + S	HMD	Time, Path deviation	Formal	48 (24)
Benko and Feiner, 2007	Object selection	O + S	HMD	Time, Error/Accuracy, Rating	Formal	12 (2)
Benko et al., 2014	NA	O + S	S/LS	Time, Error/Accuracy, Rating	Formal	11 (5)
Boring et al., 2010	Mobile AR	O + S	HH	Time, Error/Accuracy,	Formal	12 (4)
				Docking offset, subjective feedback		
Boring et al., 2011	AR control of large displays	S	HH	Discussions	Field	15 (5)
Choi and Kim, 2013	Spatial AR	O	HH, S/LS	Time, Number of clicks	Formal	13 (4)
Chun and Höllerer, 2013	Handheld AR	O + S	HH	Time, Error/Accuracy, Rating	Formal	30 (23)
Datcu and Lukosch, 2013	Basic AR interaction	O + S	HMD	Time, Error/Accuracy,	Formal	25 (9)
	with CV tracked hands			Rating, Discussions		
Denning et al., 2014	None	S	HMD	Interview analysis	Field	31 (13)
Dierker et al., 2009	None	O + S	HMD	Time, Error/Accuracy, Rating	Field	22 (11)
Fröhlich et al., 2006	Spatial information appliances	S	HH	Rating, discussion	Field	12 (5)
Grasset et al., 2012	None	S	HH	User preference	Pilot	7 (4)
Gupta et al., 2012	NA	O + S	DT	Time, Error/Accuracy, Interviews	Formal	16 (8)
Hürst and Van Wezel, 2013	Gesture-based interaction	O + S	HH	Time, Rating, Interview	Formal	21 (5)
	for phone-based AR					
Ha and Woo, 2010	Object manipulation for tangible UIs	O + S	DT	Time, Rating	Formal	20 (5)
Henderson and Feiner, 2008	AR Affordances for user interaction.	O + S	HMD	Time, Error/ Accuracy, Rating	Formal	15 (4)
Henrysson et al., 2005b	Handheld AR Interaction	O + S	HH	Time, Rating	Formal	9 (2)
Henrysson et al., 2007	Mobile AR	O + S	HH	Time, Rating	Formal	12.5 (1.5)
Henze and Boll, 2010b	NA	O + S	HH	Time, NASA TLX	Formal	12 (4)
Hoang and Thomas, 2010	Object manipulation	O + S	HMD	Time, Error/Accuracy, Rating	Formal	16 (2)
Jo et al., 2011	Selection of objects around the user	O + S	HH	Time, Error/Accuracy, Task load	Formal	16 (5)
Jones et al., 2013	None	S	S/LS	Rating, Simulator sickness	Formal	12.5 (2)
Kerr et al., 2011	Outdoor wearable AR	S	HMD	Rating	Pilot	8 (NA)
Ko et al., 2013	Handheld AR	S	HH	Rating	Formal	20 (10)
Kron and Schmidt, 2005	Telepresence	S	HMD	Rating	Formal	20 (0)
Langlotz et al., 2013	Spatialized audio in AR	S	HH	Rating	Pilot	30 (8)
Lee and Billinghurst, 2008	Multimodal interaction technique	O + S	HH, DT	Freq. of speech and gesture commands	Formal	12 (2)
Lee et al., 2009	None	O	HMD	Number of collisions with virtual wire	Formal	14 (5)
Lee et al., 2010	NA	O	HMD	No. of collisions in path tracking task	Formal	13.5 (4)
Lee and Billinghurst, 2011	Handheld outlining of AR objects	O	HH	Time, Error/Accuracy	Formal	8 (3)
Lee et al., 2013b	Spatial Interaction	S	DT	Rating	Formal	10 (2)
Lee et al., 2013c	Multimodal (speech-gesture) interaction	O + S	DT	Time, Error/Accuracy, Rating	Formal	25 (3)
Lehtinen et al., 2012	Interaction in Mobile AR	O + S	HH	Time, percieved mental workload	Formal	17 (7)
Leithinger et al., 2013	None	O	Optical-ST DT	Time	Formal	10 (4)
Looser et al., 2007	Tabletop AR; Object Selection	O + S	HMD	Time, Error/Accuracy, Rating	Formal	16 (1)
Lv, 2013	Mobile AR	S	HH	Rating	Formal	15 (6)
Maier et al., 2011	None	O	HMD	Error/Accuracy	Formal	24 (NA)
Mossel et al., 2013b	None	O + S	HH	Time, Rating, No. of steps to do task	Formal	28 (12)
Mossel et al., 2013a	3D Interaction in AR	O + S	HH	Time, Error/Accuracy, Rating	Formal	28 (12)
Mulloni et al., 2013	AR tracking initialization/calibration	O + S	HH	Error/Accuracy, Rating	Formal	7 (2)
Ofek et al., 2013	None	O + S	S/LS	Time, Error/Accuracy,	Formal	48 (26)
				Number of word detection		
Oh and Hua, 2007	Multi-display AR/VR systems	O + S	HMD, HH,	Time, Rating	Formal	9 (3)
			S/LS			
Olsson and Salo, 2011	Mobile AR	O + S	HH	Usage information	Field	90 (15)
Olsson and Salo, 2012	Mobile AR	S	None	Rating	Formal	90 (15)
Porter et al., 2010	Spatial AR	O + S	S/LS	Time, Rating	Formal	24 (5)
Pusch et al., 2008	Haptic AR	O + S	HMD	Error/Accuracy, Rating, Ranking	Formal	13 (4)
Pusch et al., 2009	Haptics	O + S	HMD	Rating, hand motion, perceived force	Formal	13 (4)
Robertson et al., 2007	None	O + S	HMD	Time, Error/Accuracy, NASA TLX	Formal	26 (12)
Robertson et al., 2008	Basic AR placement task	O + S	HMD	Time, Error/Accuracy, Rating	Formal	28 (16)
Rohs et al., 2009b	None	O + S	HH	Time, Error/Accuracy,	Formal	16.5 (10)
				Rating, motion traces, gaze shifts		
Rohs et al., 2011	Mobile AR, selection task	O	HH	Time	Formal	12 (6)
Sodhi et al., 2012	Guidance for gestures	O + S	S/LS	Time, Error/Accuracy, Rating	Formal	10 (2)
Sukan et al., 2012	Handheld AR	O + S	HH	Time, Error/Accuracy,	Pilot	15 (5.5)
				Intersection Location		
Takano et al., 2011	NA	O	HMD, DT	Error/Accuracy	Formal	15 (3)
Thomas, 2007	Mobile AR	O + S	HMD, HH	Time, Error/Accuracy, Rating	Formal	25 (5)
Toyama et al., 2014b	None	O	HMD	Error/Accuracy	Pilot	9 (5)
Toyama et al., 2014a	None	O	HMD	Error/Accuracy	Formal	10 (5)
Voida et al., 2005	Object manipulation	S	S/LS	Subjective preference	Formal	9 (6)
Weichel et al., 2014	3D printing	O + S	Non-AR	Rating, Type of gesture	Formal	11 (5.5)
White et al., 2007	None, AR Interaction Technique	S	HMD	Rating	Pilot	7 (4)
White et al., 2009	NA	O + S	HMD	Time, Error/Accuracy, Rating	Formal	13 (1)
Wither et al., 2007	None	O + S	HMD, HH	Time, Error/Accuracy, Rating	Formal	21 (4)

*S, Subjective; O, Objective; DT, Desktop; HH, handheld. Participant numbers are absolute values and where more than one studies were reported in the paper we used average counts*.

#### 4.5.1. Representative paper

Boring et al. (2010) presented a user study for remote manipulation of content on distant displays using their system, which was named Touch Projector and was implemented on an iPhone 3G. This paper received the highest ACC (31) in the Interaction category of papers. They implemented multiple interaction methods on this application, e.g., manual zoom, automatic zoom, and freezing. The user study involved 12 volunteers (four females) and was designed as a within-subjects study. In the experiment, participants selected targets and dragged targets between displays using the different conditions. Both quantitative and qualitative data (informal feedback) were collected. The main dependent variables were task completion time, failed trials, and docking offset. They reported that participants achieved highest performance with automatic zooming and temporary image freezing. This is a typical study in the AR domain based within a controlled laboratory environment. As usual in interaction studies, a significant amount of the study was focused on user performance with different input conditions, and this paper shows the benefit of capturing different types of performance measures, not just task completion time.

#### 4.5.2. Discussion

User interaction is a cross-cutting focus of research, and as such, does not fall neatly within an application category, but deeply influences user experience in all categories. The balance of expressiveness and efficiency is a core concept in general human-computer interaction, but is of even greater importance in AR interaction, because of the desire to interact while on the go, the danger of increased fatigue, and the need to interact seamlessly with both real and virtual content. Both qualitative and quantitative evaluations will continue to be important in assessing usability in AR applications, and we encourage researchers to continue with this approach. It is also important to capture as many different performance measures as possible from the interaction user study to fully understand how a user interacts with the system.

### 4.6. Medicine

One of the most promising areas for applying AR is in medical sciences. However, most of the medical-related AR papers were published in medical journals rather than the most common AR publication venues. As we considered all venues in our review, we were able to identify 43 medical papers reporting AR studies and they in total reported 54 user studies. The specific topics were diverse, including laparoscopic surgery, rehabilitation and recovery, phobia treatment, and other medical training. This application area was dominated by desktop displays (34 studies), while 16 studies used HMDs, and handheld displays were used in only one study. This is very much expected, as often in medical setups, a clear view is needed along with free hands without adding any physical load. As expected, all studies were performed in indoor locations. Thirty-six studies were within-subjects and 11 were between-subjects. The median number of participants was 13, and approximately only 14.2% of participants were females, which is considerably lower than the gender-ratio in the profession of medicine. Twenty-two studies collected only objective data, 19 collected only subjective data, and 13 studies collected both types of data. Besides time and accuracy, various domain-specific surveys and other instruments were used in these studies as shown in Table 10.

**Table 10:** Summary of user studies in Medical application areas.

**References**	**Topic**	**Data type**	**Displays used**	**Dependent measures**	**Study type**	**Participants (female)**
Akinbiyi et al., 2006	Surgery	O	TV screen	Time, Error/Accuracy,	Formal	9 (3)
				Number of broken structure, Applied force		
Albrecht et al., 2013	Medical Education	O + S	HH	Rating, Number of correct answers to exam	Formal	10 (4)
Anderson et al., 2013	Movement training	O	S/LS	Error/Accuracy	Formal	8 (2)
Archip et al., 2007	Image-guided surgery	O	DT	Error/Accuracy	Field	8 (1)
Bai et al., 2013b	Autism	O + S	S/LS	Time, Rating, video analysis of type of play	Formal	12 (2)
Bichlmeier et al., 2007	Image-Guided Surgery	O + S	DT	Time, Error/Accuracy, discussion, qualitative	Formal	12 (2)
Botden et al., 2007	Surgical Simulation	O + S	DT	Rating, surgical effetiveness measures	Formal	90 (NA)
Botden et al., 2008	Laparoscopic surgery	S	DT	Rating	Formal	55 (6)
Botella et al., 2005	Psychology/Phobia	S	HMD	Rating, user behavior	Formal	1 (1)
Botella et al., 2010	Phobia therapy	O + S	HMD	Rating	Formal	6 (6)
Botella et al., 2011	Phobia treatment	O	DT	Rating, Anxiety Disorders Interview Schedule,	Field	1 (1)
				Behavioral avoidance test,		
				Fear of spiders questionnaire,		
				Spider phobia beliefs questionnaire,		
				Subjective units of discomfort scale		
Bretón-López et al., 2010	Phobia	S	HMD	Rating	Formal	6 (6)
Brinkman et al., 2012	Laparosicpic surgical training	O	DT	Time	Formal	36 (NA)
Chintamani et al., 2010	Teleoperation	O	DT	Time, Error/Accuracy, Path Distance:	Formal	13.5 (2.5)
				Deviation From Path: Distance From Receptacle:		
Dixon et al., 2011	Image-guided surgical planning	O + S	Laparascope	Error/Accuracy	Formal	12 (NA)
Espay et al., 2010	Rehabilitation/training,	O + S	HMD	Rating, gait	Field	13 (7)
	Gait assistance for			performance		
	Parkinson's disease					
Fichtinger G. et al., 2005	Medical	O	DT	Error/Accuracy	Pilot	NA (NA)
Fichtinger G. D. et al., 2005	Medical	O	DT	Error/Accuracy	Pilot	NA (NA)
Grasso et al., 2013	Medicine	O	DT	Time, Number of scans, Dose	Field	3 (NA)
Horeman et al., 2012	Laparoscopic Training	O	DT	Time, Force applied	Pilot	12 (NA)
Horeman et al., 2014	Surgical Training	O	DT	Time, Error/Accuracy,	Formal	25 (18)
				Path length, motion volume		
Jeon et al., 2012	Medical Training	O + S	DT	Time, Similarity score	Formal	12 (2)
Juan and Prez, 2010	Phobia Treatment	S	HMD	Rating, SUS	Formal	20 (4)
Juan and Joele, 2011	Phobia	S	HMD	Rating	Formal	24 (6)
King et al., 2010	Medicine	O + S	DT	Fugl-Meyer Assessment,	Formal	4 (NA)
				Wolf Motor Function Test,		
				DASH questionnaire		
Leblanc et al., 2010	Medical Training	O + S	DT	Rating	Formal	34 (NA)
Lee et al., 2013d	Medical procedure training	O + S	DT	Error/Accuracy, Rating	Formal	40 (NA)
Luo et al., 2005b	Medical AR	O	HMD	Grasping force	Pilot	1 (0)
Luo et al., 2005a	Stroke rehabilitation	O	HMD	Clinical measures related to	Field	3 (0)
				hand grasping performance		
Markovic et al., 2014	Artificial Limbs	O	HMD	Time, Error/Accuracy, Rating	Formal	13 (NA)
Nicolau et al., 2005	Medicine	O	DT	Time, Error/Accuracy	Formal	2 (NA)
Nilsson and Johansson, 2007	Cognitive System Engineering	S	HMD	Rating	Field	12 (NA)
Regenbrecht et al., 2011	Stroke recovery and rehabilitation	O + S	DT	Time, Error/ Accuracy	Formal	64 (10)
Regenbrecht et al., 2012	Medical rehabilitation	S	DT	Rating, Discussion	Formal	36.2 (5.7)
Regenbrecht et al., 2014	AR for rehabilitation	S	DT	Rating, Interview	Formal	44 (8)
Ritter et al., 2007	Laparoscopic surgery	O	DT	path length, smoothness	Formal	60 (NA)
Teber et al., 2009	Laparoscopic Surgery	O	HMD	Time, Error/Accuracy	Field	1 (NA)
Thomas et al., 2010	Anatomical Education	S	DT	Rating	Formal	34 (21)
Wacker et al., 2006	Medical AR	O	HMD	Error/Accuracy	Formal	1 (NA)
Wilson et al., 2013	Medical procedures	O	HMD	Time, Error/Accuracy	Formal	34 (22)
Wrzesien et al., 2013	Therapy	S	HMD	Standard therapy questionaires	Formal	22 (NA)
Yoo et al., 2013	Health, Medicine	O	HMD	Rating, Balance (Berg Balance Scale, BBS),	Formal	21 (21)
				gait parameters (velocity, cadence, step length,		
				and stride length), and falls efficacy		
Yudkowsky et al., 2013	Medical Training	O + S	DT	Ability to complete medical task	Formal	16 (NA)

*S, Subjective; O, Objective; DT, Desktop; HH, handheld. Participant numbers are absolute values and where more than one studies were reported in the paper we used average counts*.

The keywords used by authors suggest that AR-based research was primarily used in *training* and *simulation*. *Laparoscopy, rehabilitation*, and *phobia* were topics of primary interest. One difference between the keywords used in medical science vs. other AR fields is the omission of the word *user*, which indicates that the interfaces designed for medical AR were primarily focused on achieving higher precision and not on user experience. This is understandable as the users are highly trained professionals who need to learn to use new complex interfaces. The precision of the interface is of utmost importance, as poor performance can be life threatening.

#### 4.6.1. Representative paper

Archip et al. (2007) reported on a study that used AR visualization for image-guided neurosurgery, which received the highest ACC (15.6) in this category of papers. Researchers recruited 11 patients (six females) with brain tumors who underwent surgery. Quantitative data about alignment accuracy was collected as a dependent variable. They found that using AR produced a significant improvement in alignment accuracy compared to the non-AR system already in use. An interesting aspect of the paper was that it focused purely on one user performance measure, alignment accuracy, and there was no qualitative data captured from users about how they felt about the system. This appears to be typical for many medical related AR papers.

#### 4.6.2. Discussion

AR medical applications are typically designed for highly trained medical practitioners, which are a specialist set of users compared to other types of user studies. The overwhelming focus is on improving user performance in medical tasks, and so most of the user studies are heavily performance focused. However, there is an opportunity to include more qualitative measures in medical AR studies, especially those that relate to user estimation of their physical and cognitive workload, such as the NASA TLX survey. In many cases medical AR interfaces are aiming to improve user performance in medical tasks compared to traditional medical systems. This means that comparative evaluations will need to be carried out and previous experience with the existing systems will need to be taken into account.

### 4.7. Navigation and driving

A total of 24 papers reported 28 user studies in the Navigation and Driving application areas (see Table 11). A majority of the studies reported applications for car driving. However, there were also pedestrian navigation applications for both indoors and outdoors. Fifteen studies used handheld displays, five used HMDs, and two used heads-up displays (HUDs). Spatial or large-screen displays were used in four studies. Twenty-three of the studies were performed in controlled setups and the remaining five were executed in the field. Twenty-two studies were designed as within-subjects, three as between-subjects, and the remaining three were mixed-factors studies. Approximately 38% of participants were females in these studies, where the median number of participants used was 18. Seven studies were performed in an outdoor environment and the rest in indoor locations. This indicates an opportunity to design and test hybrid AR navigation applications that can be used in both indoor and outdoor locations. Seven studies collected only objective data, 18 studies collected a combination of both objective and subjective data, whereas only three studies were based only on subjective data. Task completion time and error/accuracy were the most commonly used dependent variables. Other domain specific variables used were headway variation (deviation from intended path), targets found, number of steps, etc.

**Table 11:** Summary of user studies in Navigation and Driving application area.

**References**	**Topic**	**Data type**	**Displays used**	**Dependent measures**	**Study type**	**Participants (female)**
Arning et al., 2012	AR navigation systems	O + S	HH, S/LS	Rating, Navigation performance metrics	Formal	24 (19)
Avery et al., 2008	Outdoor AR navigation	O + S	HMD	Time, Error/Accuracy, Rating	Formal	34 (9)
Choi et al., 2011	Outdoor AR (Mobile)	O + S	HH	Time, Rating, Clicks on	Formal	12 (1)
				screen, number targets found		
Dünser et al., 2012a	Outdoor navigation	O + S	HH	Time, Rating, Discussion; video coding	Formal	22 (11)
Fröhlich et al., 2011	In-car navigation using AR	O + S	“in-car” screen	Rating, See measures.	Formal	31 (11)
Gee et al., 2011	Cooperative AR,	O + S	HH	Time, Error/Accuracy	Formal	12 (2)
	Automatic map building					
Goldiez et al., 2007	Navigation	O	HMD	Time, Percentage of maze covered	Formal	120 (60)
Ha et al., 2012	Path editing using	O + S	HMD	Time, Error/Accuracy,	Formal	16.5 (1)
	tangible user interfaces			Rating		
Heller et al., 2014	Navigation	O	HH	Path, orientation	Formal	16.5 (2.5)
Kjeldskov et al., 2013	Mobile urban map-based info.	O + S	HH	Rating, Interviews	Field	58 (6)
Möller et al., 2012	Indoor navigation	S	DT	Rating	Formal	81 (39)
Möller et al., 2014	Navigation	O + S	HH	Time, Error/Accuracy, Rating	Formal	12 (1)
Morrison et al., 2009	AR Map, Augmenting a paper map	S	HH	Rating, Primarily an observational study	Field	37 (20)
Moussa et al., 2012	AR for driving analysis	O + S	HMD	Error/Accuracy	Formal	44 (18)
Mulloni et al., 2011a	Indoor navigation	O	HH	Time, Error/Accuracy, steps	Formal	10 (5)
Mulloni et al., 2011b	Navigation	O + S	HH	Time, Rating, where AR was used	Field	9 (NA)
Ng-Thow-Hing et al., 2013	Automotive Augmented Reality	O	HUD on car	Error/Accuracy	Formal	16 (8)
		O	windshield			
Rohs et al., 2007	Moble maps on handheld display	O + S	HH	Time, Error/Accuracy, Rating	Formal	18 (10)
Rohs et al., 2009a	Map navigation	O	HH	Time, Error/Accuracy	Formal	17 (12)
Rusch et al., 2013	Driving	O	S/LS	Time, Error/Accuracy	Formal	27 (14)
Schall et al., 2013b	Driving	O	Projection	Time, Error/Accuracy, Response Rate,	Formal	20 (7)
		O	HUD	Time to collision, Headway variation		
Tönnis et al., 2005	Driving	O + S	S/LS	Time, Error/Accuracy, Rating	Formal	12 (2)
Tönnis and Klinker, 2007	Driving	O + S	HUD	Time, Error/Accuracy, Rating	Formal	24 (10)
Tangmanee and Teeravarunyou, 2012	Vehicle Navigation	O + S	S/LS	Time, Subjective questions,	Formal	5 (2)
				number of eye fixations		

*S, Subjective; O, Objective; DT, Desktop; HH, handheld. Participant numbers are absolute values and where more than one studies were reported in the paper we used average counts*.

Analysis of author-specified keywords suggests that *mobile* received a strong importance, which is also evident by the profuse use of handheld displays in these studies, since these applications are about mobility. *Acceptance* was one of the noticeable keywords, which indicates that the studies intended to investigate whether or not a navigation interface is acceptable by the users, given the fact that, in many cases, a navigational tool can affect the safety of the user.

#### 4.7.1. Representative paper

Morrison et al. (2009) published a paper reporting on a field study that compared a mobile augmented reality map (MapLens) and a 2D map in a between-subjects field study, which received the highest ACC (16.3) in this application area of our review. MapLens was implemented on a Nokia N95 mobile phone and use AR to show virtual points of interest overlaid on a real map. The experimental task was to play a location-based treasure hunt type game outdoors using either MapLens or a 2D map. Researchers collected both quantitative and qualitative (photos, videos, field notes, and questionnaires) data. A total of 37 participants (20 female) took part in the study. The authors found that the AR map created more collaborations between players, and argued that AR maps are more useful as a collaboration tool. This work is important, because it provides an outstanding example of an AR Field study evaluation, which is not very common in the AR domain. User testing in the field can uncover several usability issues that normal lab-based testing cannot identify, particularly in the Navigation application area. For example, Morrison et al. (2009) were able to identify the challenges for a person of using a handheld AR device while trying to maintain awareness of the world around themselves.

#### 4.7.2. Discussion

Navigation is an area where AR technology could provide significant benefit, due to the ability to overlay virtual cues on the real world. This will be increasingly important as AR displays become more common in cars (e.g., windscreen heads up displays) and consumers begin to wear head mounted displays outdoors. Most navigation studies have related to vehicle driving, and so there is a significant opportunity for pedestrian navigation studies. However human movement is more complex and erratic than driving, so these types of studies will be more challenging. Navigation studies will need to take into consideration the user's spatial ability, how to convey depth cues, and methods for spatial information display. The current user studies show how important it is to conduct navigation studies outdoors in a realistic testing environment, and the need to capture a variety of qualitative and quantitative data.

### 4.8. Perception

Similar to Interaction, Perception is another general field of study within AR, and appears in 51 papers in our review. There were a total of 71 studies reported in these papers. The primary focus was on visual perception (see Table 12) such as perception of depth/distance, color, and text. A few studies also reported perception of touch (haptic feedback). AR X-ray vision was also a common interface reported in this area. Perception of egocentric distance received significant attention, while exocentric distance was studied less. Also, near- to medium-field distance estimation was studied more than far-field distances. A comprehensive review of depth perception studies in AR can be found in Dey and Sandor (2014), which also reports similar facts about AR perceptual studies as found in this review.

**Table 12:** Summary of user studies in Perception application area.

**References**	**Topic**	**Data type**	**Displays used**	**Dependent measures**	**Study type**	**Participants (female)**
Blum et al., 2010	General AR	S	HMD, DT	Rating	Formal	18 (4)
Dey et al., 2010	X-ray vision	O + S	HH	Time, Error/Accuracy,	Formal	20 (2)
				Rating, NASA TLX		
Dey et al., 2012	X-ray vision	O + S	HH	Error/Accuracy	Formal	20 (NA)
Gabbard et al., 2005	Outdoor AR	O	HMD	Time, Error/Accuracy	Formal	18 (6)
Gabbard et al., 2006	Perception	O	HMD	Time, Error/Accuracy	Formal	18 (6)
Gabbard et al., 2007	Text legibility	O	HMD	Time, Error/Accuracy	Formal	24 (12)
Gabbard and Swan II, 2008	Outdoor AR	O	HMD	Time, Error/Accuracy	Formal	24 (12)
Gandy et al., 2010	AR Testbed Design	O + S	HMD	Error/Accuracy, Physiological measures	Formal	20 (6)
Grechkin et al., 2010	Distance estimation	O	HMD	Distance walked	Formal	53.5 (23.5)
Gustafsson and Gyllenswärd, 2005	Ambient Displays	S	Ambient display	interview questions	Pilot	15 (4)
Hincapié-Ramos et al., 2014	None	S	HH	Interview questions	Pilot	8 (2)
Iwai et al., 2013	Spatial AR	O + S	S/LS	Time, Error/Accuracy	Formal	10 (1)
Jankowski et al., 2010	Text readability	O + S	DT	Time, Error/Accuracy, Rating	Formal	20 (4)
Jeon and Choi, 2011	Haptic rendering of stiffness	S	Phantom	Psychophysical PSE	Formal	12 (4)
Jeon and Harders, 2012	Haptic AR	S	HMD, Phantom	Rating	Pilot	6 (2)
Jones et al., 2008	None	O	HMD	Error/Accuracy	Formal	NA (NA)
Jones et al., 2011	None	O	HMD	Error/Accuracy	Formal	21.75 (NA)
Kellner et al., 2012	None	O	HMD	Time, Error/Accuracy	Formal	14.5 (6)
Kerber et al., 2013	None	O	HH	Error/Accuracy	Formal	12 (2)
Kim, 2013	Context in handheld AR	S	HH, HH projectors	Rating	Field	20 (10)
Knörlein et al., 2009	None	O	HMD	Correct selection of	Formal	14 (7)
				strongest force		
Lee et al., 2012	AR haptic perception	O + S	DT	Rating, Perceived location	Formal	14 (5)
Lee et al., 2013a	NA	O + S	HMD	Time, Rating	Formal	48 (28)
Lindeman et al., 2007	None	O	AudioBone bone-	Error/Accuracy, Frequency	Formal	24 (2)
			conducting headset			
Liu et al., 2010	Displays	O + S	HMD	Error, Rating	Formal	10 (2)
Liu et al., 2012	Handheld AR	O	HH	Time, Error/Accuracy	Formal	16 (4)
Livingston et al., 2005	NA	O + S	HMD	Error/Accuracy	Formal	8 (NA)
Livingston, 2007	Visual acuity in AR displays	O	HMD, DT	Time, Error/Accuracy	Formal	5 (1)
Livingston and Ai, 2008	Tracking error	O + S	HMD	Time, Error/Accuracy, Rating	Formal	11 (1)
Livingston et al., 2009c	Basic visual perception	O	HMD	Time, Error/Accuracy	Formal	20 (5.5)
Livingston et al., 2009b	Basic perception in AR	O	HMD	Time, Error/Accuracy	Formal	11 (2)
Livingston et al., 2009a	Object depth perception	S	HMD	Time, Error/Accuracy, Rating	Formal	12 (4)
Livingston et al., 2011	Military situation awareness	O	HMD	Error/Accuracy	Formal	14 (3)
Lu et al., 2012	Visual search	O	DT	Time, Error/Accuracy	Formal	20.5 (7)
Mercier-Ganady et al., 2014	None	O + S	S/LS	Rating, BCI ouput	Formal	12 (NA)
Olsson et al., 2012	Mobile AR	S	None	Rating	Formal	262 (133)
Peterson et al., 2009	None	O + S	Projection HUD	Time, Error/Accuracy, Rating	Formal	16 (NA)
Pucihar et al., 2014	None	O + S	HH	Time, Error/Accuracy, Subject preference	Formal	15 (4)
Salamin et al., 2006	Unspecified	S	HMD	Rating, Able to perform tasks	Pilot	6 (0)
Sandor et al., 2010	X-Ray Vision	O + S	HH	Time, Rating	Formal	21.5 (1)
Singh et al., 2010	NA	O	HMD	Error/Accuracy, Distance to object	Formal	18 (7)
Singh et al., 2012	Depth Perception	O	HMD	Error/Accuracy	Formal	40 (NA)
Suzuki et al., 2013	None	O + S	HMD	Rating, Cardio-visual and tactile-visual feedback modulate proprioceptive drif,	Formal	21 (11)
Tomioka et al., 2013	User-perspective cameras	O	HH	Time	Pilot	9.3 (0.7)
Tsuda et al., 2005	See-through vision	S	HH	Rating	Formal	14 (0)
Veas et al., 2011	Mobile AR	S	DT	Rating	Formal	18.6 (5.3)
Veas et al., 2012	Outdoor topography	S	HH	Comments/Feedback	Heuristic	7.5 (1)
Wagner et al., 2006	3D Characters in AR	O + S	HH	Error/Accuracy, Rating	Formal	13 (4)
Wither and Höllerer, 2005	Distance estimation	O + S	HMD	Rating, Judged Depth	Formal	19 (5)
Wither et al., 2011	Mobile AR	O	HH	Error/Accuracy	Field	13.5 (0)
Zhang et al., 2012	Depth perception	O	HMD	Error/Accuracy	Formal	52 (NA)

*S, Subjective; O, Objective; DT, Desktop; HH, handheld. Participant numbers are absolute values and where more than one studies were reported in the paper we used average counts*.

Twenty-one studies used handheld displays, 34 studies used HMDs, and 9 studies used desktop displays. The Phantom haptic display was used by two studies where haptic feedback was studied. Sixty studies were performed as controlled lab-based experiments, and only three studies were performed in the field. Seven studies were pilot studies and there was one heuristic study (Veas et al., 2012). Fifty-three studies were within-subjects, 12 between-subjects, and six mixed-factors. Overall, the median number of participants used in these studies was 16, and 27.3% of participants were females. Fifty-two studies were performed in indoor locations, only 17 studies were executed outdoors, and two studies used both locations. This indicates that indoor visual perception is well studied whereas more work is needed to investigate outdoor visual perception. Outdoor locations present additional challenges for visualizations such as brightness, screen-glare, and tracking (when mobile). This is an area to focus on as a research community. Thirty-two studies were based on only objective data, 14 used only subjective data, and 25 studies collected both kinds of data. Time and error/accuracy were most commonly used dependent measures along with subjective feedback.

Keywords used by authors indicate an emphasis on *depth* and *visual* perception, which is expected, as most of the AR interfaces augment the visual sense. Other prominent keywords were *X-ray* and *see-through*, which are the areas that have received a significant amount of attention from the community over the last decade.

#### 4.8.1. Representative paper

A recent paper by Suzuki et al. (2013), reporting on the interaction of exteroceptive and interoceptive signals in virtual cardiac rubber hand perception, received the highest ACC (13.5) in this category of papers. The authors reported on a lab-based within-subjects user study using 21 participants (11 female) who wore a head-mounted display and experienced a tactile feedback simulating cardiac sensation. Both quantitative and qualitative (survey) data were collected. The main dependent variables were proprioceptive drift and virtual hand ownership. Authors reported that ownership of the virtual hand was significantly higher when tactile sensation was presented synchronously with the heart-beat of the participant than when provided asynchronously. This shows the benefit of combing perceptual cues to improve the user experience.

#### 4.8.2. Discussion

A key focus of AR is trying to create a perceptual illusion that the AR content is seamlessly part of the user's real world. In order to measure how well this is occurring it is important to conduct perceptual user studies. Most studies to date have focused on visual perception, but there is a significant opportunity to conduct studies on non-visual cues, such as audio and haptic perception. One of the challenges of such studies is being able to measure the users perception of an AR cue, and also their confidence in how well they can perceive the cue. For example, asking users to estimate the distance on an AR object from them, and how sure they are about that estimation. New experimental methods may need to be developed to do this well.

### 4.9. Tourism and exploration

Tourism is one of the relatively less explored areas of AR user studies, represented by only eight papers in our review (Table 13). A total of nine studies were reported, and the primary focus of the papers was on museum-based applications (five papers). Three studies used handheld displays, three used large-screen or spatial displays, and the rest head mounted displays. Six studies were conducted in the field, in the environment where the applications were meant to be used, and only three studies were performed in lab-based controlled environments. Six studies were designed to be within-subjects. This area of studies used a markedly higher number of participants compared to other areas, with the median number of participants being 28, with approximately 38% of them female. All studies were performed in indoor locations. While we are aware of studies in this area that have been performed in outdoor locations, these did not meet the inclusion criteria of our review. Seven studies were based completely on subjective data and two others used both subjective and objective data. As the nature of the interfaces were primarily personal experiences, the over reliance on subjective data is understandable. An analysis of keywords in the papers found that the focus was on *museums*. *User* was the most prominent keyword among all, which is very much expected for an interface technology such as AR.

**Table 13:** Summary of user studies in Tourism and Exploration application area.

**References**	**Topic**	**Data type**	**Displays used**	**Dependent measures**	**Study type**	**Participants (female)**
Alvarez-Santos et al., 2014	Human-robot interaction, Tourism	O + S	DT	Error/Accuracy, Rating	Formal	12 (NA)
Asai et al., 2010	Interaction for museum exhibit	S	S/LS	Rating	Field	155 (NA)
Baldauf et al., 2012	AR for public displays	S	HH, S/LS	Rating	Field	31 (15)
Hatala et al., 2005	Museums	S	Headphones	Rating	Field	6 (NA)
Olsson et al., 2013	Mobile AR	S	HH	Interview responses	Field	28 (16)
Pescarin et al., 2012	Museums	S	Unspecified	Comments from interviews,	Field	362 (199)
		S		questionnaire		
Sylaiou et al., 2010	Museums	S	S/LS	Rating	Formal	29 (13)
Tillon et al., 2011	Museums	S	HH	Rating	Field	16 (NA)

*S, Subjective; O, Objective; DT, Desktop; HH, handheld. Participant numbers are absolute values and where more than one studies were reported in the paper we used average counts*.

#### 4.9.1. Representative paper

The highest ACC (19) in this application area was received by an article published by Olsson et al. (2013) about the expectations of user experience of mobile augmented reality (MAR) services in a shopping context. Authors used semi-structured interviews as their research methodology and conducted 16 interview sessions with 28 participants (16 female) in two different shopping centers. Hence, their collected data was purely qualitative. The interviews were conducted individually, in pairs, and in groups. The authors reported on: (1) the characteristics of the expected user experience and, (2) central user requirements related to MAR in a shopping context. Users expected the MAR systems to be playful, inspiring, lively, collective, and surprising, along with providing context-aware and awareness-increasing services. This type of exploratory study is not common in the AR domain. However, it is a good example of how qualitative data can be used to identify user expectations and conceptualize user-centered AR applications. It is also an interesting study because people were asked what they expected of a mobile AR service, without actually seeing or trying the service out.

#### 4.9.2. Discussion

One of the big advantages of studies done in this area is the relatively large sample sizes, as well as the common use of “in the wild” studies, that assess users outside of controlled environments. For these reasons, we see this application area as useful for exploring applied user interface designs, using real end-users in real environments. We also think that this category will continue to be attractive for applications that use handheld devices, as opposed to head-worn AR devices, since these are so common, and get out of the way of the content when someone wants to enjoy the physically beautiful/important works.

## 5. Conclusion

### 5.1. Overall summary

In this paper, we reported on 10 years of user studies published in AR papers. We reviewed papers from a wide range of journals and conferences as indexed by Scopus, which included 291 papers and 369 individual studies. Overall, on average, the number of user study papers among all AR papers published was less than 10% over the 10-year period we reviewed. Our exploration shows that although there has been an increase in the number of studies, the relative percentage appears the same. In addition, since 2011 there has been a shift toward more studies using handheld displays. Most studies were formal user studies, with little field testing and even fewer heuristic evaluations. Over the years there was an increase in AR user studies of educational applications, but there were few collaborative user studies. The use of pilot studies was also less than expected. The most popular data collection method involved filling out questionnaires, which led to subjective ratings being the most widely used dependent measure.

### 5.2. Findings and suggestions

This analysis suggests opportunities for increased user studies in collaboration, more use of field studies, and a wider range of evaluation methods. We also find that participant populations are dominated by mostly young, educated, male participants, which suggests the field could benefit by incorporating a more diverse selection of participants. On a similar note, except for the Education and Tourism application categories, the median number of participants used in AR studies was between 12 and 18, which appears to be low compared to other fields of human-subject research. We have also noticed that within-subjects designs are dominant in AR, and these require fewer participants to achieve adequate statistical power. This is in contrast to general research in Psychology, where between-subject designs dominate.

Although formal, lab-based experiments dominated overall, the Education and Tourism application areas had higher ratios of field studies to formal lab-based studies, which required more participants. Researchers working in other application areas of AR could take inspiration from Education and Tourism papers and seek to perform more studies in real-world usage scenarios.

Similarly, because the social and environmental impact of outdoor locations differ from indoor locations, results obtained from indoor studies cannot be directly generalized to outdoor environments. Therefore, more user studies conducted outdoors are needed, especially ethnographic observational studies that report on how people naturally use AR applications. Finally, out of our initial 615 papers, 219 papers (35%) did not report either participant demographics, study design, or experimental task, and so could not be included in our survey. Any user study without these details is hard to replicate, and the results cannot be accurately generalized. This suggests a general need to improve the reporting quality of user studies, and education of researchers in the field on how to conduct good AR user studies.

### 5.3. Final thoughts and future plans

For this survey, our goal has been to provide a comprehensive account of the AR user studies performed over the last decade. We hope that researchers and practitioners in a particular application area can use the respective summaries when planning their own research agendas. In the future, we plan to explore each individual application area in more depth, and create more detailed and focused reviews. We would also like to create a publicly-accessible, open database containing AR user study papers, where new papers can be added and accessed to inform and plan future research.

## Author contributions

All authors contributed significantly to the whole review process and the manuscript. AD initiated the process with Scopus database search, initial data collection, and analysis. AD, MB, RL, and JS all reviewed and collected data for an equal number of papers. All authors contributed almost equally to writing the paper, where AD and MB took the lead.

### Conflict of interest statement

The authors declare that the research was conducted in the absence of any commercial or financial relationships that could be construed as a potential conflict of interest.
